# Long-Chain Fatty Acid Combustion Rate Is Associated with Unique Metabolite Profiles in Skeletal Muscle Mitochondria

**DOI:** 10.1371/journal.pone.0009834

**Published:** 2010-03-24

**Authors:** Erin L. Seifert, Oliver Fiehn, Véronic Bezaire, David R. Bickel, Gert Wohlgemuth, Sean H. Adams, Mary-Ellen Harper

**Affiliations:** 1 Department of Biochemistry, Microbiology and Immunology, University of Ottawa, Ottawa, Ontario, Canada; 2 Genome Center, University of California Davis, Davis, California, United States of America; 3 Ottawa Institute for Systems Biology, University of Ottawa, Ottawa, Ontario, Canada; 4 Obesity and Metabolism Research Unit, USDA-ARS Western Human Nutrition Research Center, Agricultural Research Service, United States Department of Agriculture, and Department of Nutrition, University of California Davis, Davis, California, United States of America; Instituto de Química - Universidade de São Paulo, Brazil

## Abstract

**Background/Aim:**

Incomplete or limited long-chain fatty acid (LCFA) combustion in skeletal muscle has been associated with insulin resistance. Signals that are responsive to shifts in LCFA β-oxidation rate or degree of intramitochondrial catabolism are hypothesized to regulate second messenger systems downstream of the insulin receptor. Recent evidence supports a causal link between mitochondrial LCFA combustion in skeletal muscle and insulin resistance. We have used unbiased metabolite profiling of mouse muscle mitochondria with the aim of identifying candidate metabolites within or effluxed from mitochondria and that are shifted with LCFA combustion rate.

**Methodology/Principal Findings:**

Large-scale unbiased metabolomics analysis was performed using GC/TOF-MS on buffer and mitochondrial matrix fractions obtained prior to and after 20 min of palmitate catabolism (n = 7 mice/condition). Three palmitate concentrations (2, 9 and 19 µM; corresponding to low, intermediate and high oxidation rates) and 9 µM palmitate plus tricarboxylic acid (TCA) cycle and electron transport chain inhibitors were each tested and compared to zero palmitate control incubations. Paired comparisons of the 0 and 20 min samples were made by Student's t-test. False discovery rate were estimated and Type I error rates assigned. Major metabolite groups were organic acids, amines and amino acids, free fatty acids and sugar phosphates. Palmitate oxidation was associated with unique profiles of metabolites, a subset of which correlated to palmitate oxidation rate. In particular, palmitate oxidation rate was associated with distinct changes in the levels of TCA cycle intermediates within and effluxed from mitochondria.

**Conclusions/Significance:**

This proof-of-principle study establishes that large-scale metabolomics methods can be applied to organelle-level models to discover metabolite patterns reflective of LCFA combustion, which may lead to identification of molecules linking muscle fat metabolism and insulin signaling. Our results suggest that future studies should focus on the fate of effluxed TCA cycle intermediates and on mechanisms ensuring their replenishment during LCFA metabolism in skeletal muscle.

## Introduction

Long-chain fatty acids (LCFA) are a crucial energy source in mammalian peripheral tissues, including skeletal muscle. During fasting physiological suppression of glucose uptake and oxidation in muscle is an important feature of the shift to greater FA oxidation (FAO) rate [1], enabling glucose to be spared for cells that have an obligatory requirement for that substrate. Suppressed glucose uptake and oxidation can, however, develop despite plentiful glucose supply, leading eventually to the development of type 2 diabetes (T2DM) [2]. Insulin resistance in skeletal muscle has important consequences in this regard because this tissue contributes substantially to whole body insulin-mediated glucose disposal.

It is well established that FAs and other lipid species can modulate signaling pathways in skeletal muscle, including insulin signaling [2], [3]. With the exceptions of the diacylglycerides and ceramides, the mechanisms through which lipid species can impair insulin signaling remain poorly understood [4], [5], [6], [7], [8]. In particular, the relationship between mitochondrial FA uptake/oxidation and insulin sensitivity in muscle is controversial. Individuals with T2DM have lower skeletal muscle FAO rates in the fasted state [9], [10], as well as decreased subsarcolemmal mitochondrial content [11], decreased expression of oxidative phosphorylation genes [12], [13], and decreased maximal NADH-linked phosphorylating respiration in isolated mitochondria [14], [15]. First degree relatives of T2DM patients also exhibited reduced muscle mitochondrial content [16] and impaired switching to FAO and mitochondrial adaptation to a high-fat diet [17]. In rats, overexpression of carnitine palmitoyltransferase 1, a key controlling enzyme in FAO, at physiologic levels improved insulin sensitivity [18]. These findings have suggested that impaired FAO (and mitochondrial function) promotes insulin resistance. Yet other studies using pharmacological, dietary or genetic interventions have linked insulin resistance in muscle to increased FAO rates [19], [20], [21], [22], [23]. Moreover, improved insulin sensitivity was correlated with lower FAO rate despite higher intramuscular long-chain acyl-CoA species [22]. Thus a shift from lipid oxidation to storage does not necessarily promote insulin resistance. It has been proposed that insulin resistance is associated with excessive flux through β-oxidation relative to tricarboxylic acid cycle (TCA) capacity [22], [24], a phenomenon termed mitochondrial FA overload [22].

Collectively, the above raises the possibility that metabolites associated with mitochondrial FA utilization are important in determining insulin action in muscle. However, beyond the acyl-carnitine species [22], [25], [26], [27], the metabolites associated with skeletal muscle mitochondrial FAO are poorly explored. Here, we have utilized the novel technology of global metabolomics in combination with a unique experimental design to identify metabolite signatures of LCFA combustion in skeletal muscle mitochondria oxidizing palmitate at different rates. This experimental approach allowed the intra- and extra-mitochondrial localization of metabolites, along with determinations of altered pathway flux. Ultimately, the identification of muscle mitochondrial metabolites may reveal candidate molecules and/or pathways linking mitochondrial FA handling and lipid-mediated modulation of insulin signaling.

## Methods

### Animals and Reagents

Female C57Bl/6J mice (n = 7) were obtained from Jackson Laboratories at 4 months of age, housed in our facility at 23°C (12∶12 hr light cycle, lights on 0700), with free access to chow (4.5% fat/weight; Charles River-5075), for at least 1 week before being studied. Animals were cared for in accordance with the principles and guidelines of the Canadian Council on Animal Care and the Institute of Laboratory Animal Resources (National Research Council). This study was approved by the Animal Care Committee of the University of Ottawa. Unless otherwise stated, reagents were from Sigma (Oakville, ON, Canada).

### Isolation of Mitochondria from Skeletal Muscle

Skeletal muscle mitochondria were isolated essentially according to Chappell and Perry [28]. All media were ice-cold, and procedures done on ice or at 4°C. Briefly, pectoral, forelimb and hindlimb muscles were rapidly dissected and placed in basic medium [BM (mM): KCl (140), HEPES (20), MgCl_2_ (5), EGTA (2); pH 7.0]. Together, these muscle groups comprise a mixed population of mainly type II oxidative and glycolytic fibers. Muscle was cleaned of connective tissue and fat, minced and placed in 15 vol of homogenizing medium (HM: BM with 1 mM ATP and 1% BSA (w/v)) containing one unit of protease (Subtilisin A) per g muscle wet weight. Tissue was homogenized using a glass/Teflon Potter-Elvehjem tissue grinder (240 rpm) and fractionated by centrifugation at 800 g (10 min), and the supernatant collected and spun at 12000 g (9 min). The pellet was resuspended in 20 ml BM and incubated on ice for 5 min (myofibrillar repolymerization). Samples were spun at 800 g (8 min) to pellet actin-myosin polymers. The supernatant was then spun at 12000 g (9 min). The final pellet was resuspended in 220 µl of BM. This isolation procedure yields mitochondria with high respiratory control ratios (state 3/state 4; ∼8–10 when supplied with 10 mM pyruvate/5 mM malate), and which are capable of activating palmitate [29], a process dependent on the integrity of enzymes on the mitochondrial outer membrane. Protein concentration was determined by a modified Lowry method with BSA as standard.

### Incubation Conditions

Each of the 7 mitochondrial preparations derived from independent mice was tested in all treatment conditions. Mitochondria (0.6 mg/ml) were supplied with three concentrations of palmitate corresponding to low (2 µM), medium (9 µM) and high (19 µM) rates of β-oxidation [29]. Three ml aliquots of incubation medium [IM, (mM): KCl (120), HEPES (5), KH_2_PO_4_ (5), MgCl_2_ (5) and EGTA (1); pH 7.4] were supplemented with (mM) ATP (1), malate (0.05), coenzyme A (0.025), and carnitine (0.5) and added to 20-ml glass reaction vials. Solutions of low, medium and high palmitate concentrations were added to vials in a 6∶1 FA:BSA complex. Palmitate was solubilized in ethanol; the final concentration of ethanol in the reaction mixture was 0.5%. Two additional incubations were performed as controls. The first control condition (0 µM palmitate) evaluated the metabolic profile of mitochondria oxidizing only malate, and included ATP, carnitine and CoA and ethanol (0.5%). The second control condition (9 µM palmitate + inhibitors) assessed effects of FA in the absence of complete oxidative catabolism, and consisted of malate, 9 µM palmitate, ATP, carnitine and CoA, and supplemented with the TCA cycle inhibitor malonate (10 mM) and the electron transport chain complex I inhibitor rotenone (5 µM) [30].

### Experimental Design

**Figure 1:**
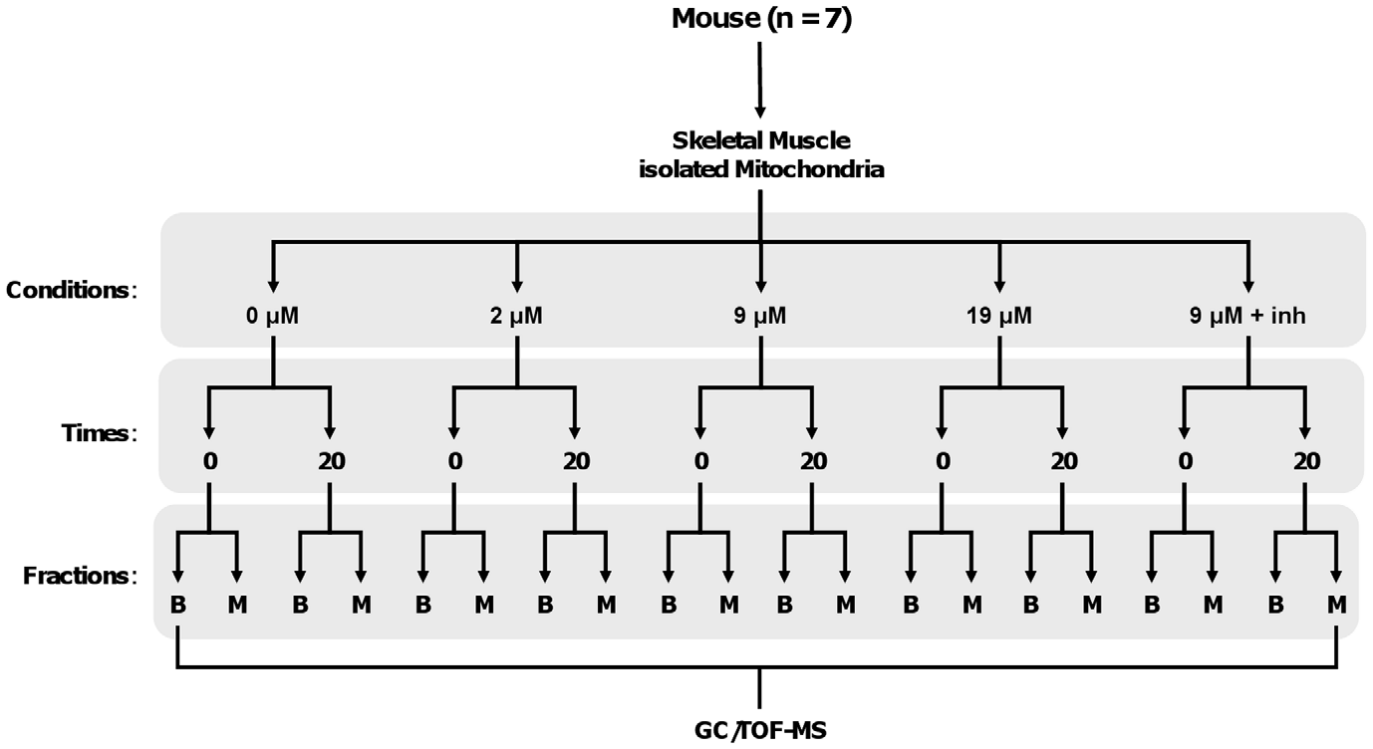
Summary of experimental design used to determine metabolite shifts in murine muscle mitochondrial preparations under different rates of LCFA β-oxidation. Conditions: incubations with/without palmitate, in the absence or presence of inhibitors (inh; 10 mM malonate, 5 µM rotenone). Times: subsamples of mitochondrial suspension removed at 0 and 20 min for subsequent metabolomics determinations. Fractions: mitochondrial suspensions were separated into matrix (M) and buffer (B). GC/TOF-MS: gas chromatography, time-of-flight mass spectroscopy.

Figure 1 summarizes the experimental design. Each mitochondrial preparation (n = 7) was tested in each of the five conditions described above: 0, 2, 9, and 19 µM palmitate, and 9 µM palmitate + inhibitors. Vials were pre-warmed for 5 min at 37°C, and mitochondria were added to initiate the reaction. Immediately upon addition of mitochondria, half of each reaction mixture was transferred to tubes submerged in an ice slurry (‘time 0 min’ fraction); the time 0 fraction remained in the ice slurry for ∼2 minutes prior to centrifugation (see below). The second half was incubated for 20 min at 37°C, and the reaction stopped on ice as for the time 0 min fraction (‘time 20 min’ fraction). This timed sampling enabled later estimation of flux. Our choice of the 20-min time point is based on pilot experiments showing that the rate of CO_2_ production was similar with a 20 or 60 min incubation.

At each time point (time 0 and 20 min), aliquots were separated into mitochondrial and buffer compartments by centrifugation (12000 g, 3 min, 4°C). A 1300-µl aliquot of the incubation medium (‘buffer’ fraction) was transferred to an empty tube, flash-frozen in liquid N_2_, then stored at −80°C; removal of only a fraction of the buffer ensured that the mitochondrial pellet was not disrupted. The remaining incubation medium was carefully removed and discarded, with care taken to remove as much of the medium as possible with minimal disruption of the pellet. Pellets were resuspended in 100 µl of IM without supplementation. A series of six freeze-thaw cycles (liquid N_2_ and 37°C) was used to disrupt mitochondrial membranes. Membranes were pelleted at 20000 g (10 min, 4°C) and 90 µl of the supernatant was collected (‘matrix’ fraction).

### Metabolite Identification

Matrix and buffer samples were supplemented with 13 internal standards for retention index correction (C8-C30 fatty acid methyl esters) as previously reported [31]. Samples were subsequently dried down and derivatized with 2 µl of a solution of 20 mg/ml of 98% pure methoxyamine hydrochloride in pyridine at 30°C for 90 min to protect aldehyde and ketone groups. 8 µl of MSTFA with 1% TMCS (Pierce, Rockford IL) was added for trimethylsilylation of acidic protons at 37°C for 30 min. 1 µl of this mixture was injected by a Gerstel automatic liner exchange system and CIS cold injection system (Gerstel, Muehlheim, Germany). For every 10 samples, a fresh multibaffled liner was inserted (Gerstel #011711-010-00) using the Maestro1 Gerstel software (version 1.1.4.18). Before and after each injection, the 10 µl injection syringe was washed three times with 10 µl ethyl acetate. The injector was operated in splitless mode, opening the split vent after 25 s. Chromatography was performed on an Agilent 6890 gas chromatograph (Santa Clara CA), under control of the Leco ChromaTOF software (version 2.32) (St. Joseph MI), using a 30 m long, 0.25 mm i.d. Rtx-5Sil MS column with 0.25 µm 95% dimethyl 5% diphenyl polysiloxane film and an additional 10 m integrated guard column (Restek, Bellefonte PA). Helium carrier gas was set at a constant flow of 1 ml/min with an oven temperature ramp starting at 50°C for 1 min and then ramped at 20°C/min to 330°C at which point it was held constant for 5 min. Mass spectra were acquired using a Leco Pegasus IV time of flight mass spectrometer with a transfer line temperature of 280°C and electron impact ionization at 70 V under a mass resolving power R = 600 from m/z 85–500 at 10 spectra s^−1^ and 1850 V detector voltage. The instrument performed autotuning for mass calibration using FC43 (perfluorotributylamine) and a 4-point calibration using a quality control mixture of 30 known metabolites before starting analysis sequences. ChromaTOF (version 2.32) was used for data preprocessing without smoothing, 3 s peak width, baseline subtraction just above the noise level, automatic mass spectral deconvolution and peak detection at signal/noise levels of 10∶1 throughout the chromatogram. Result *.txt files were exported to a data server with absolute spectra intensities and further processed by the BinBase algorithm [32] for removing inconsistent and noisy spectra. Metabolites were identified in BinBase and its underlying Fiehn mass spectral library using retention index windows of ± 2,000 units (around ± 2 s retention time deviation) and mass spectral similarity thresholds that varied upon signal/noise ratios and mass spectral purity values as given in supplemental data of Fiehn *et al*. [31].

### Data Analysis

Metabolite values were log transformed, and subsequent analysis was performed on these values. Data are presented as log-transformed; log transformation of metabolite data can generally be taken as normally distributed [33], justifying the use of parametric analysis. Each incubation condition was tested in each mitochondrial preparation, therefore paired analyses were performed. Thus, for each metabolite, comparison between the 0 and 20 min values for each metabolite was made by paired Student's t-test. To account for multiple comparisons, each false discovery rate (FDR) was estimated by the product of the significance level (Type I error rate) and the number of null hypotheses tested divided by the number of null hypotheses rejected; this estimator is conservative [34], [35], [36]. Outlier analysis was performed using Grubbs' test. A single outlier was removed in each of only 11 data sets; this is flagged for the affected comparisons. Unless otherwise indicated, values are presented as the mean ± SEM.

## Results

### Optimization of Flux Conditions

Mitochondria were supplied with three concentrations of palmitate corresponding to low (2 µM), medium (9 µM) and high (19 µM) rates of FAO, as determined previously using mitochondria isolated and incubated under identical conditions as in the current study, but tracking 1-[^14^C]-palmitate [29]. Over this range of palmitate concentration, flux of C16∶0 carboxyl carbon to CO_2_ (*i.e.* complete oxidation) leveled off with increasing palmitate concentration whereas incomplete oxidation, measured as the ^14^C-label recovered in the acid soluble product of a lipid extraction, increased linearly; thus there was a disproportionate increase in incomplete relative to complete FAO (Figure S1). These observations suggested that metabolite profiles of mitochondria oxidizing palmitate at high *versus* low rates may differ substantially, providing insight into how different LCFA oxidation rates impact mitochondrial function and signaling from mitochondria. We therefore determined metabolite profiles of mitochondria oxidizing palmitate at low, medium and high rates. Two control conditions were also assessed. To determine metabolite shifts under basal conditions, the first control treatment contained all incubation constituents except FA. The second control incubation assessed the specificity of metabolite shifts observed with alterations in FAO via inclusion of FAO inhibitors along with 9 µM palmitate.

To assess the bioenergetic status of mitochondria oxidizing palmitate, O_2_ consumption was measured under the incubation conditions used to generate samples for metabolomics analyses. As presented in Table S1 (and explained in the accompanying text), palmitate is oxidized under phosphorylating conditions, at a rate between state 3 and state 4 but closer to state 3.

Metabolite profiling of the mitochondrial matrix and buffer fractions enabled metabolite shifts between compartments to be determined. In the matrix fraction, a decrease in metabolite concentration indicates that mitochondrial catabolism of the metabolite was increased and/or that the metabolite was effluxed into the buffer fraction; insight into one or the other of these interpretations can be gained by comparing changes in matrix and buffer concentrations of a particular metabolite. Regarding an increase in the concentration of a matrix metabolite, a distinction cannot be made between an increase in a metabolite due to its net synthesis *versus* one caused by a decrease in its degradation. A further possible interpretation is an accumulation of a metabolite due to its release from membrane-associated sites as could occur if related enzymes exist as an organized, membrane-bound complex. In the buffer fraction, an increase in the concentration of a metabolite indicates efflux from the mitochondrial fraction, whereas a reduction in concentration indicates mitochondrial uptake.

### Global Characteristics of the Metabolomics Data Set

**Figure 2:**
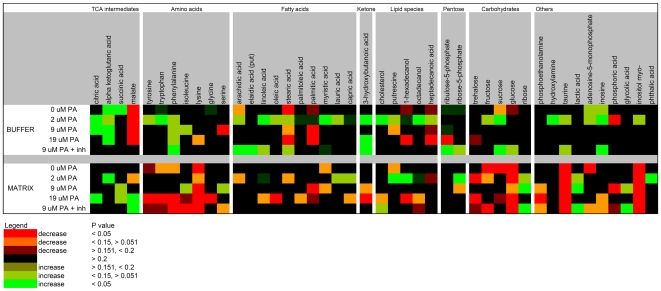
Heat map showing the identified metabolites from murine muscle mitochondrial preparations that changed in concentration over the course of the incubations. Metabolites are shown for which the concentration changed significantly during the 20-min incubation for one or more of the treatment conditions (0, 2, 9, and 19 µM palmitate, and 9 µM palmitate plus FAO inhibitors).

**Figure 3:**
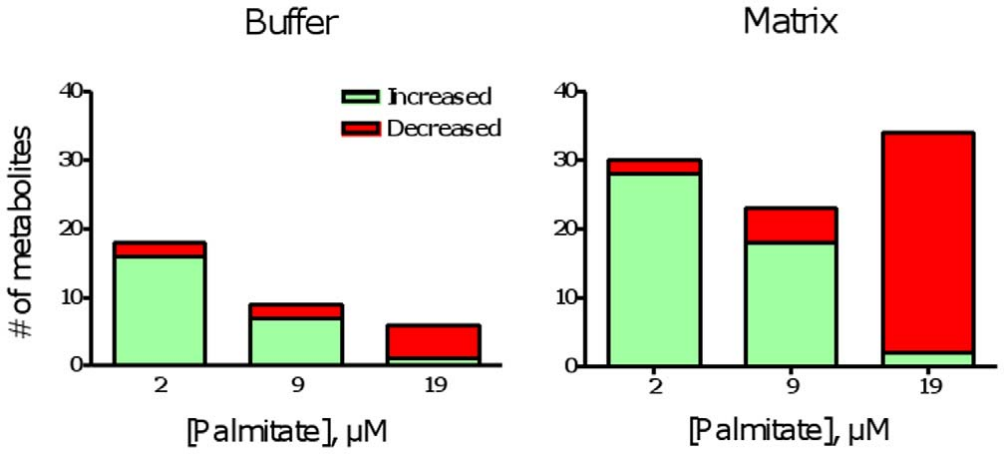
Numbers of metabolites that changed significantly in concentration (P<0.05, paired t-test) in buffer and matrix fractions obtained from skeletal muscle mitochondria oxidizing LCFA at different rates.

The global characteristics of the metabolomics data set are summarized in Tables 1 and 2, and Figures 2 and 3. Importantly, only high-quality metabolic signals were reported after applying the multi-tiered filtering algorithm implemented in the BinBase database [31]. Approximately half of all detected signals per chromatogram are not reported in the final data set due to these stringent quality control procedures. For example, each reported metabolite must be confirmed with high mass spectral similarity and at the correct retention index in at least 80% of the samples in at least one of the treatment classes of the experimental design [31]. This leads to a very conservative assessment of the number of identified metabolites that nevertheless spans various chemical classes from medium and long-chain free fatty acids and steroids to organic phosphates, polar and non-polar amino acids and hydroxyl acids (Table 1). Raw and processed data sets can be downloaded from SetupX, detailing the total of 197 analytes that were reliably detected after BinBase processing and annotating the 51 metabolites that were unambiguously identified by PubChem and KEGG database identifiers. All metabolites were further characterized by the ion trace used for quantification, the retention index, the BinBase identifier and the full mass spectrum encoded as a string in the result tables (Table S2 and downloads from SetupX [http://fiehnlab.ucdavis.edu:8080/m1/main_public.jsp]).

**Table 1:** Global characteristics of metabolomics dataset.

	Number of Observations
Total number of detected metabolites	197
Number of unambiguously identified metabolites	51
**Chemical Classes**	
Fatty acid species	15
Organic acids	10
Amino acids	8
Carbohydrates	5
Phosphates	4
Alcohols	3
Nucleotides	2
Amines	2
Steroids	1
Inorganic ions	1

**Table 2:** Estimated false discovery rates (FDR), P value threshold (paired t-test), and corresponding number of metabolites (#) that changed over the 20-min incubation in each fraction (matrix, buffer).

	Condition	FDR, %	P value	#	FDR, %	P value	#	FDR, %	P value	#	FDR, %	P value	#
**Matrix**	**0 **µ**M**	31	0.05	32	23	0.035	30	19	0.02	20	19	0.005	5
	**2 **µ**M**	21	0.05	48	16	0.035	42	14	0.02	28	11	0.005	9
	**9 **µ**M**	28	0.05	35	20	0.035	30	18	0.02	18	14	0.005	7
	**19 **µ**M**	21	0.05	46	17	0.035	41	14	0.02	33	9	0.005	11
	**9 **µ**M+inh**	21	0.05	50	17	0.035	40	13	0.02	31	7	0.005	15
**Buffer**	**0 **µ**M**	25	0.05	36	23	0.035	20	19	0.02	21	14	0.005	7
	**2 **µ**M**	32	0.05	31	28	0.035	23	33	0.02	12	16	0.005	6
	**9 **µ**M**	75	0.05	13	48	0.035	13	48	0.02	8	33	0.005	3
	**19 **µ**M**	70	0.05	14	76	0.035	7	46	0.02	7	20	0.005	3
	**9 **µ**M+inh**	33	0.05	30	34	0.035	20	26	0.02	15	16	0.005	6

Condition: concentration of palmitate; inh: inhibitors added.

False discovery rate (FDR) estimates, P value threshold and numbers of metabolites for each FDR and P values are presented in Table 2. Due to stringent quality control filters which minimized the number of reported metabolites, and the use of a conservative FDR estimator, a P value threshold of 0.05 was selected for presentation and discussion of results despite the high FDR estimates for some conditions.

Global representations of the data set are presented as a heat map (Figure 2) as well as in Table S2. The heat map depicts only identified metabolites that changed significantly in at least one condition over the 20-min incubation. Intensity and color are based on the degree of significance and direction of change, respectively. Degree of significance was utilized to emphasize the robust time-dependent changes in the data set. Some patterns, including patterns that are unique to FAO rate, are apparent from the heat map. As a group, TCA cycle intermediates (TCAi) were found to accumulate in both the matrix and the buffer; the pattern of change depended on the condition (see below). Amino acids tended to decrease in the matrix, and did so most robustly with 19 µM palmitate (see below). Fatty acids also tended to decrease in the matrix at high FAO rate, but tended to accumulate in the buffer fraction at the lowest FAO rate as well as in the presence of inhibitors.

The numbers of metabolite shifts (0 *vs.* 20 min, P<0.05) uniquely associated with FAO was a function of both experimental condition and compartment (buffer or matrix) (Figure 3). Of interest was the extent to which the number and direction of the shifts differed among the three palmitate oxidation conditions. A large number of metabolites accumulated with 2 µM palmitate in both the matrix (27 metabolites) and the buffer (16 metabolites). In contrast, oxidation of 19 µM palmitate was associated with a large reduction in the number of matrix metabolites (32 metabolites) whereas only one and two metabolites increased significantly in the buffer and matrix respectively. The number of metabolites that changed with 9 µM palmitate was intermediate between that with high and low FAO rates. This overview of the data set indicates that palmitate oxidation rate *per se* is associated with distinct metabolite changes in both the matrix and buffer compartments.

### Shifts in Selected Metabolites

**Figure 4:**
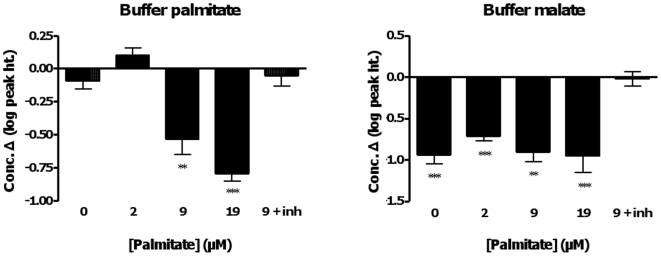
Change in the concentration of supplied substrates (palmitate, malate) in the buffer fraction obtained from murine muscle mitochondria over the course of the incubations. All 5 conditions are shown (0, 2, 9 and 19 µM palmitate, and 9 µM palmitate plus inhibitors). Values are the log of the change in concentration (peak height). Significance: paired t-test, 0 *vs*. 20 min, within treatment; * P<0.05; **, P<0.02, *** P<0.005.

As a validation of our methodology, we examined the changes in palmitate and malate in the buffer fraction (Figure 4). As expected, there was a significant reduction over the course of the 20 min reaction in the concentration of palmitate in the buffer with medium (70±10% decrease; P = 0.02) and high (85±5% decrease; P<0.0001) palmitate supply, and no significant change in palmitate concentration when inhibitors were present. Complete and incomplete oxidation were measurable for the 2 µM palmitate incubation (see Figure S1); however, a change in palmitate could not be detected at this low palmitate supply. The buffer was similarly depleted of malate, except when inhibitors were present, as expected.

Some metabolites changed over time in all or in 4 of 5 conditions, in the same direction and by a similar magnitude, indicating that these changes were not specific for any of the treatment conditions. This occurred for 4 metabolites in the matrix fraction (taurine, inositol, glucose, and unidentified analyte 219881) and illustrates the utility of the control conditions.

Among the metabolites identified by our unbiased approach, the significant changes that were the most prominent in terms of magnitude as well as showing a pattern related to FAO were the TCAi, amino acids, and a ketone body species (3-hydroxybutanoic acid).

#### TCA cycle intermediates

**Figure 5:**
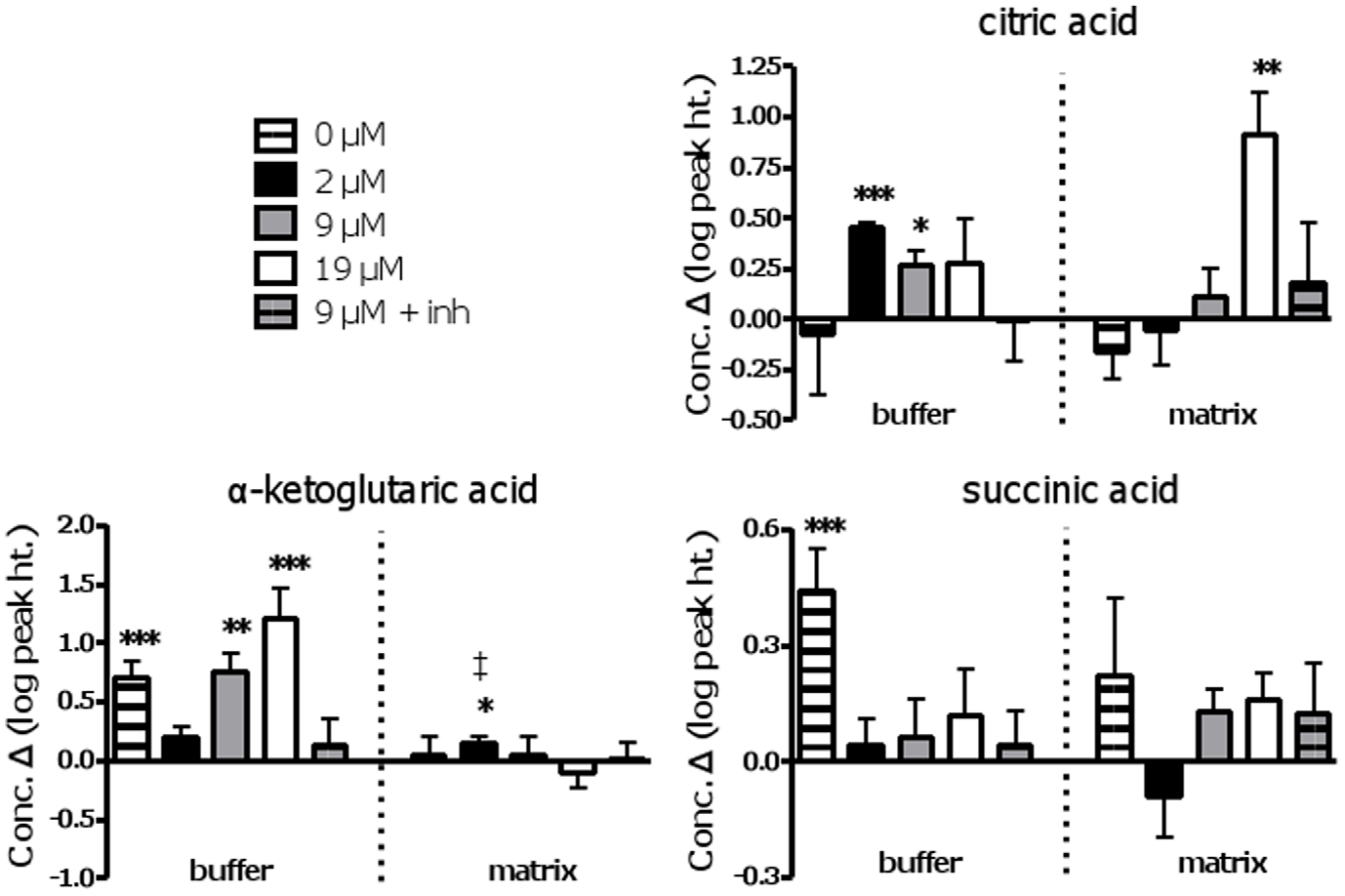
Changes in detected TCA cycle intermediates over time in buffer and matrix fractions obtained from muscle mitochondrial preparations. All 5 conditions are shown (0, 2, 9 and 19 µM palmitate, and 9 µM palmitate plus inhibitors) for both fractions (matrix and buffer). Values are the log of the change in concentration (peak height) over a 20-min incubation. Buffer and matrix fractions can be compared within each metabolite. Significance: paired t-test, 0 *vs*. 20 min, within treatment; * P<0.05; **, P<0.02, *** P<0.005. ‡: indicates that an outlier was removed from either the 0 or 20 min group for that metabolite in that fraction.

The detected TCAi were malate, citrate, α-ketoglutarate (α-KG) and succinate. While significant changes were observed in the matrix fraction, the most pronounced changes were found in the buffer fraction. Citrate accumulated in the buffer at the low (P = 0.0007; ∼130% increase) and medium (P = 0.03; ∼30% increase) but not at the highest palmitate concentration (Figure 5). Corresponding changes in citrate were not detected in the matrix fraction, indicating citrate production in the matrix during the 20 min incubation with subsequent efflux. Export of citrate depended on substrate oxidation since export was not detected with TCA cycle and electron transport chain inhibition. In contrast, a large rise in matrix citric acid was measured with oxidation of 19 µM palmitate (P<0.001; ∼800% increase), but interestingly there was no significant export of this metabolite to the buffer.

Matrix levels of α-KG showed little change, with the exception of a small increase with the lowest palmitate oxidation rate (P<0.05). α-KG was found to accumulate in the buffer fraction with increasing palmitate supply (P = 0.02 and 0.003 for 9 µM and 19 µM palmitate, respectively) (Figure 5). An increase was also detected in the zero palmitate control that was of a comparable magnitude to that observed with 9 µM palmitate (∼400% increase), but less than that with 19 µM palmitate (∼1400% increase). These increases in buffer α-KG in the absence of a reduction in the matrix suggest that net production of α-KG occurred during the 20 min incubation. As for citrate, export of α-KG was not detected in the presence of inhibitors. Taken with the citrate results, the data support the idea that citrate and α-KG production rose with increasing palmitate β-oxidation, with robust export of α-KG from the matrix space.

The absolute changes in matrix and buffer citrate and α-KG, as well as the time 0 values, were estimated using standard curves and the value of total extracted mitochondrial protein (∼5 mg mitochondrial protein/ gram muscle wet weight) (see Methods S1). The time 0 value for citrate of ∼60 nmol/g muscle wet weight was of the same magnitude as that reported from whole rat skeletal muscle (80–180 nmol/g muscle wet weight; [37], [38]). The reported values were, however, from mainly type I whereas our muscle contained primarily type II fibers which would be expected to contain citrate levels due to lower mitochondrial content. The time 0 value for α-KG was ∼33 nmol/g muscle wet weight, similar to that in whole mouse heart [39]. Over the course of the 20-min incubation with 19 µM palmitate, the calculated change in matrix citrate was 1.4 nmol/min/g muscle wet weight. Incubation with 2 µM palmitate resulted in net accumulation of α-KG at 1.9 nmol/min/g muscle wet weight. During incubation with 2 and 9 µM palmitate, net citrate efflux occurred at 0.4 and 0.2 nmol/min/g muscle wet weight, respectively. Compared to citrate, substantially higher net efflux rates were measured for α-KG (2.6 and 8 nmol/min/g muscle wet weight during incubation with 9 and 19 µM palmitate, respectively).

Matrix levels of succinate were not significantly changed in any of the treatment conditions (Figure 5). In the buffer, only the succinate in the zero palmitate control changed significantly, showing a ∼200% increase (P<0.005). While there was a trend for such an increase with increasing palmitate concentration, changes were not significant. Changes in malate are confounded by its addition as a substrate. However, it is noteworthy that matrix levels of malate increased significantly in the presence of inhibitors (P<0.02; ∼300% increase) as would be expected, but also increased with 19 µM palmitate (P = 0.02; ∼30% increase) (Table S2).

#### Amino acids

**Figure 6 pone-0009834-g006:**
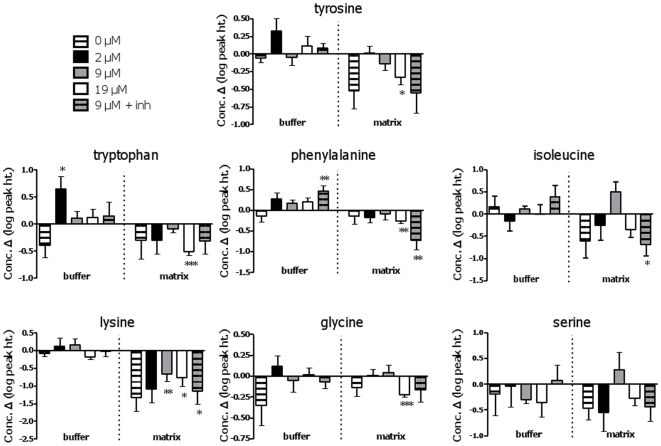
Changes in detected amino acids over time in buffer and matrix fractions obtained from muscle mitochondrial preparations. Significance: paired t-test, 0 *vs*. 20 min, within treatment; * P<0.05; **, P<0.02, *** P<0.005. See legend to Figure 5 for further details.

Seven amino acids were detected, including both polar (serine, lysine) and non-polar (glycine, phenylalanine, tyrosine, tryptophan, isoleucine) species (Figure 6). The most pronounced and consistent change was a significant reduction in the matrix levels of 5 amino acids (tyrosine, tryptophan, phenylalanine, lysine, glycine) with oxidation of 19 µM palmitate. Lysine was also reduced with 9 µM palmitate and in the presence of inhibitors; therefore this change may not be related to FAO *per se*. There was no corresponding accumulation in the buffer of any of these five species, indicating net degradation in the matrix fraction. Two species (tryptophan, phenylalanine) accumulated in the buffer in either the inhibitor condition (phenylalanine) or with 2 µM palmitate; a corresponding reduction in the matrix was observed with phenylalanine only.

#### 3-hydroxybutanoic acid (3-hydroxybutyrate; 3-OHBA)

**Figure 7:**
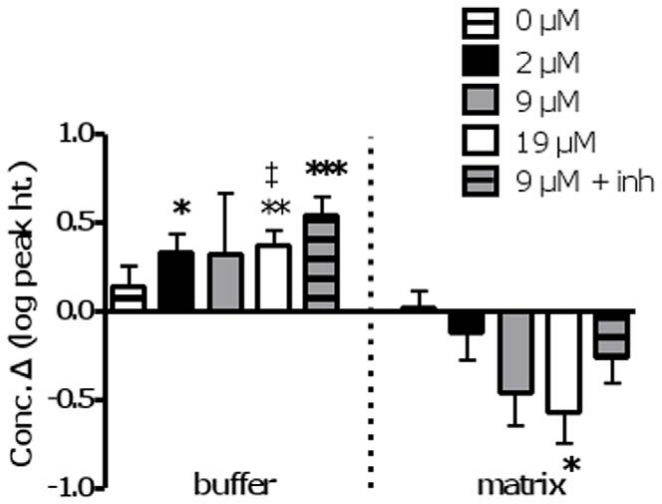
Changes in 3-hydroxybutanoic acid over time in buffer and matrix fractions obtained from muscle mitochondrial preparations. Significance: paired t-test, 0 *vs*. 20 min, within treatment; * P<0.05; **, P<0.02, *** P<0.005. ‡: indicates that an outlier was removed. See legend to Figure 5 for further details.

In the matrix fraction, a significant change in 3-OHBA was only detected with oxidation of 19 µM palmitate (Figure 7). However, 3-OHBA accumulated in the buffer in all palmitate conditions with the exception of 9 µM palmitate for which there was a large degree of variability in the sample set. For the 19 µM palmitate condition, buffer accumulation exceeded matrix reduction (+76778±22196 *vs.* -1175±316 arbitrary units). Thus, net synthesis of 3-OHBA is suggested for the inhibitor+9 µM condition and concurrent with FAO.

#### Unidentified metabolites

**Figure 8:**
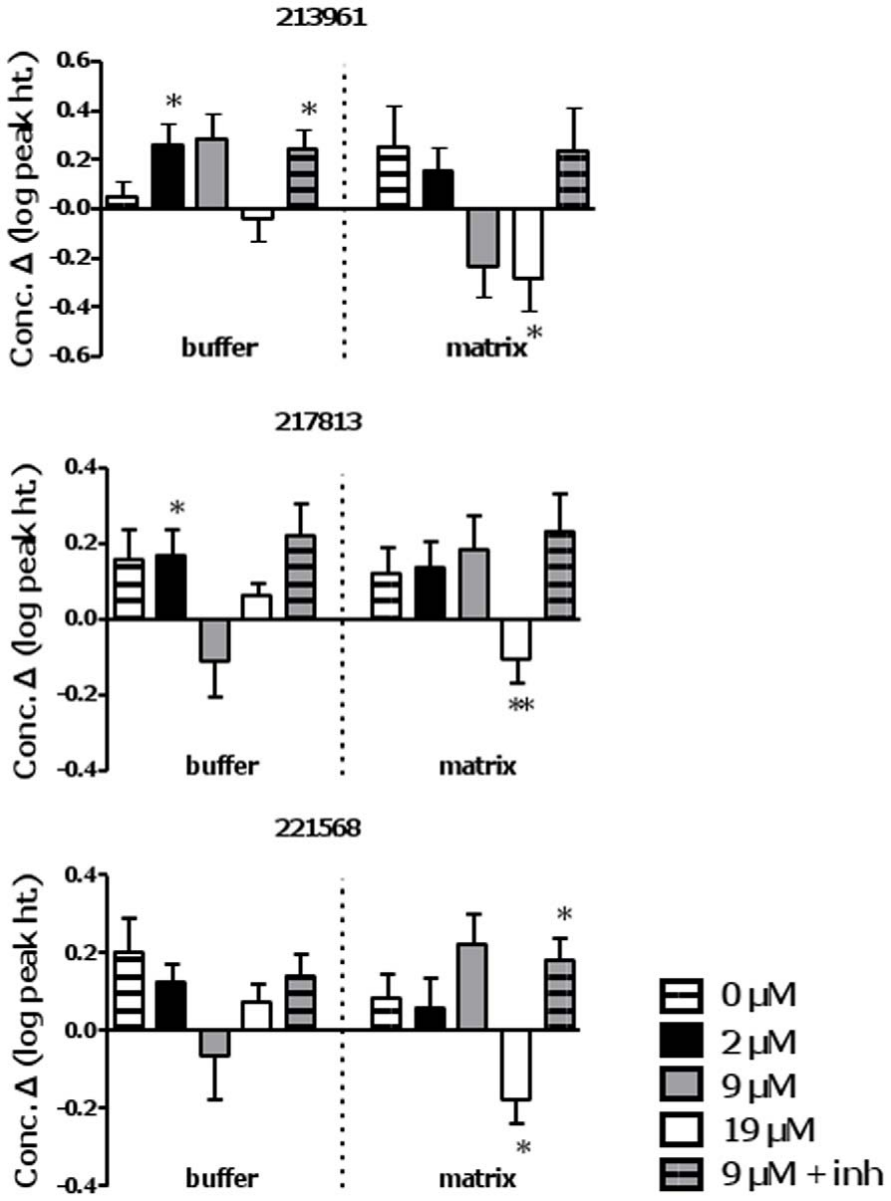
Change over time in levels of selected unidentified metabolites in buffer and matrix fractions obtained from muscle mitochondrial preparations. Significance: paired t-test, 0 *vs*. 20 min, within treatment; * P<0.05; **, P<0.02. See legend to Figure 5 for further details.

Mass spectra of unknown metabolites can be viewed and compared to other BinBase entries through a web service [http://eros.fiehnlab.ucdavis.edu:8080/binbase-compound/database/selectDatabase]. Many unknown species were detected. However, only a small subset showed significant changes specific to FAO; three such analytes are presented in Figure 8. The overall pattern of change of these 3 metabolites was matrix disappearance with 19 µM palmitate and buffer accumulation with 2 µM palmitate in the absence of a matrix reduction. Other potential species of interest are: 215355, 217840, 222047, 222099.

## Discussion

Long-chain FAs are a key carbon source in tissues that consume ATP at high rates, such as cardiac muscle and skeletal muscle during exercise or fasting. Cellular disposal of FA is associated with reduced insulin-mediated glucose uptake [2], and a role for mitochondrial FAO has been suggested [22]; however the mechanistic basis for such a role is unknown. This question is of particular interest given that β-oxidation flux may exceed TCA cycle flux [22]; in rodents, such ‘mitochondrial FA overload’ was reflected in higher incomplete oxidation, and specifically in increased serum and muscle levels of acylcarnitines [22]. Compartmentalization of these metabolites within muscle was not determined, and other potential species of interest were not identified. Here, we utilized unbiased metabolite profiling of skeletal muscle mitochondria oxidizing LCFA at different rates to provide insight into changes in mitochondrial function and intermediary metabolism during FAO. Our approach demonstrated overall that FAO and FAO rate are associated with unique shifts in mitochondrial metabolites and with the efflux of metabolites from mitochondria. Several pathways emerged as being worthy of further study, namely the TCA cycle and ketone body metabolism (Figure S2). Altogether our findings demonstrate the power and applicability of the novel experimental design, technology and analytical approaches utilized.

### TCA Cycle Anaplerosis

Our observations indicate that titration of β-oxidation impacts TCA cycle dynamics; that is, production and/or catabolism as well as efflux of TCA cycle intermediates (TCAi). Supportive evidence is summarized as follows. Changes in TCAi (citrate, α-KG, succinate, malate) were detected in the matrix and buffer in all conditions in which complete oxidation was possible (*i.e.*, in the absence of inhibitors), indicating that metabolite shifts depended on TCA cycle and/or electron transport chain flux. In the case of efflux into the buffer, passive exchange of existing pools of intermediates with malate cannot explain these observations since matrix levels of intermediates were never found to decrease. Efflux is also unlikely to reflect non-specific leakage of metabolites from mitochondria since only a small fraction of all detected metabolites increased in the buffer in any of the conditions, and efflux of TCAi was not even detected in the inhibitor condition. That the oxidizing conditions were associated with a distinct pattern of change in the TCAi is striking (Figure 5). Buffer or matrix accumulation of citrate and α-KG was apparent with FAO, and the magnitude dependent on rate. However, α-KG also accumulated in buffer with provisioning of malate alone (zero palmitate control). While speculative, the significant increase in net α-KG efflux with zero palmitate might be explained by net flux toward succinate, since under these conditions there is minimal fuel-derived acetyl-CoA to combine with oxaloacetate. In support of this view, we observed a robust net efflux of succinate only in the zero palmitate control condition. Finally, accumulation of malate in the matrix was associated only with the highest FAO rate, and α-KG accumulation in the buffer was also most apparent in this condition. Thus, FAO rate appears to influence TCA cycle dynamics. This interpretation concurs with some observations made in the isolated working heart [40], [41]; however important differences between skeletal and cardiac muscle may exist, as discussed below. Also, it is noteworthy that prior studies in skeletal muscle only measured TCAi in whole muscle extracts [37], [38], [42], [43]; thus the present study is the first to compartmentalize TCAi in skeletal muscle and to directly link mitochondrial TCAi efflux with FAO rate.

Each of the oxidizing conditions was associated with increased matrix and/or buffer levels of intermediates without a reduction in matrix levels. These observations could arise from one or a combination of the following: increased synthesis of an intermediate; its slowed catabolism; its release from membrane-associated sites. Anaplerosis of TCAi replenishes loss of carbon through metabolites other than CO_2_. Anaplerotic generation of citrate and α-KG in the present study concurs with previous reports in skeletal and cardiac muscle [37], [38], [40], [41], [42], [43]. Physiologically, the main anaplerotic substrates are pyruvate, glutamate/glutamine, and precursors of propionyl-CoA (some amino acids, odd-chain FAs, and C5-ketone bodies) [44]. Here, exogenous malate was provided as an anaplerotic substrate, and would be expected to contribute to TCAi generation. In support, malate disappeared from the buffer in all oxidizing conditions indicating its uptake by mitochondria, and, except at the highest FAO rate, it did not accumulate in the matrix indicating that it was catabolized. Possible additional anaplerotic substrates in isolated mitochondria are stored pyruvate and amino acids. It has however been suggested that isolated muscle mitochondria contain only limited stores of pyruvate [45]. We detected amino acids in the matrix fraction and, interestingly, levels tended to decrease with increased FAO, suggesting a link between FAO rate and amino acid catabolism. It needs to be recognized that of the seven detected amino acids, only two (glycine and serine) are known to undergo initial breakdown in the mitochondrial matrix. Initial catabolism of the other five is thought to occur extra-mitochondrially, with subsequent import of intermediates into the matrix. That we could detect the disappearance of four of five of these amino acids without accumulation in the buffer, and this disappearance was, with the exception of lysine, confined to the highest FAO rate, implicate a specific process that can be regulated by FAO. A possible scenario is that these amino acids underwent catabolism, and thus that related enzymes are intimately associated with skeletal muscle mitochondria, and/or that amino acids in addition to glycine and serine can be initially catabolized within the matrix.

Glycine was depleted in the matrix only at the highest FAO rate. Glycine can be degraded to pyruvate via conversion to serine. That there was no net accumulation of serine at the highest FAO rate supports the possibility that serine was degraded to pyruvate. Conversion of serine to pyruvate is believed to partially occur extra-mitochondrially; again, it is possible that related enzymes are either intimately associated with mitochondria or are present within the matrix. Pyruvate can be degraded to α-KG via alanine aminotransferase, or to citrate via carboxylation or decarboxylation. Notably, citrate and α-KG accumulated at the highest FAO rate. As well, flux through each of the latter pathways has been documented in muscle; flux through alanine aminotransferase accounted for increased α-KG in skeletal muscle at the onset of exercise [43], and, in the isolated working heart perfused with high or low concentrations of oleate, both carboxylation and decarboxylation contributed to citrate production [41]. Little is known about the role of pyruvate carboxylase activity in skeletal muscle mitochondria except that the protein is expressed, albeit at lower levels than in heart [46]. Increased oleate supply to the heart was associated with reduced anaplerotic carboxylation and decarboxylation of pyruvate [41]. Thus, at high FAO rate, pyruvate conversion to TCAi may be minimal and flux through alanine aminotransferase may become a more important anaplerotic mechanism. Our overall approach, combined with targeted metabolomics and the provision of isotopically-labelled anaplerotic substrates, could be used in focused studies of anaplerosis during FAO in skeletal muscle mitochondria.

Studies on the functional role of anaplerosis in muscle have focused mainly on the heart and demonstrated that insufficient anaplerosis leads to reduced contractile function (reviewed in [46]). Yet in exercising skeletal muscle, interventions that limited anaplerosis have not affected ATP production, even at VO_2_max [43], [47]. A role for anaplerosis in skeletal muscle is evident, however, in the context of enhanced reliance on FAs or of aberrant FAO. In patients with very long-chain acyl-dehydrogenase deficiency, the provision of anaplerotic triheptanoin relieved muscle weakness [48]. In type 2 diabetics, plasma propionylcarnitine correlated inversely with HbA1c, suggesting reduced anaplerotic capacity with type 2 diabetes severity[24]. That matrix levels of TCAi did not decrease in the present model indicates that anaplerosis likely was not limiting and was well-matched to removal of TCAi (‘cataplerosis’; *e.g*. [44]). However, if anaplerotic capacity were diminished, ongoing efflux of intermediates would lower TCA cycle capacity, reducing the oxidative disposal of LCFA-CoA. Moreover, during FAO, a reduction in pyruvate as an anaplerotic substrate may result from inhibition of mitochondrial uptake and lower anaplerotic utilization, as reported in hearts supplied with FAs [41].

### Efflux of TCA Cycle Intermediates

Citrate and α-KG were detected in the buffer fraction, indicative of efflux from skeletal muscle mitochondria. Little information exists on the efflux of TCAi from skeletal muscle mitochondria, although studies in muscle extracts suggest that it occurs [37], [38]. In the isolated working heart supplied with different concentrations of oleate, efflux of citrate, measured as citrate release from whole heart, was observed, and the rate was the same for high and low oleate concentrations [40], [41]. We found citrate efflux to be most robust at the lowest palmitate concentration, measurable at the medium concentration, but not significantly increased at the highest concentration. Thus, our observation of a lack of significant mitochondrial citrate efflux at the highest FAO rate differs from the situation in cardiac muscle. The absence of malate as a counter-molecule for citrate exchange cannot be an explanation since malate was supplied exogenously, disappeared from the buffer fraction, and a fate other than mitochondrial association seems unlikely. Competition by the tricarboxylic acid and oxoglutarate carriers for malate is a possibility. However, the Km values of the tricarboxylic acid carrier for malate and citrate are lower than the Km values of the oxoglutarate carrier for malate and α-KG [49], favouring exchange of malate for citrate. Thus the difference between heart and skeletal muscle in citrate efflux may depend on other factors such as differential modulation of citrate or α-KG transport. In heart supplied with isotopically labeled oleate, pyruvate and lactate, and in skeletal muscle supplied with glucose and insulin, effluxed citrate contributed to malonyl-CoA production [37], [38], [40]. Both pyruvate and oleate contributed to the acetyl moiety of malonyl-CoA in the heart [40]. Because our results suggest that citrate export in skeletal muscle mitochondria depends on FAO rate, and, at least at high FAO rate, to be differently modulated relative to cardiac mitochondria, further investigation into the association of malonyl-CoA with citrate and FAO rate in skeletal muscle is warranted.

### Ketone Body Production & TCA Cycle Activity

Hepatic ketone body production is a crucial source of acetyl-CoA equivalents for other tissues including skeletal muscle, and ketogenesis is a mechanism by which finite pools of matrix free CoASH are maintained and reducing equivalents consumed to support continued robust hepatic LCFA β-oxidation. Thus, much of the 3-OHBA detected in the matrix fraction at time 0 presumably originated in the liver. However, as 3-OHBA accumulated in the buffer in excess of its reduction in the matrix, we can conclude that 3-OHBA was somehow produced in the matrix. These changes in 3-OHBA occurred in the presence of inhibitors, indicating that complete palmitate oxidation to CO_2_ was not required. Ketogenesis in muscle is, however, contentious. Mitochondrial HMG CoA synthase, a key control point in ketogenesis [50], has thus far only been reported in skeletal muscle as a transcript [51]. As well, ketone body production in the heart [52], [53] has been explained by exchange between acetoacetate and acetyl-CoA pools resulting from the reversibility of the ketolytic reactions (‘pseudoketogenesis’) [53]. Thus, it is premature to propose that our findings indicate true ketogenesis. Yet, they warrant further investigation especially in light of the observed export of 3-OHBA, as well as the association of 3-OHBA with insulin resistance and reactive oxygen species formation [54], [55].

Excess acetyl-CoA units, resulting from greater β-oxidation relative to TCA cycle flux, would become available to generate 3-OHBA or acetylcarnitine [22]. Potentially adding to this mismatch is inactivation of aconitase or α-KG dehydrogenase by reactive oxygen species [56], [57], that are particularly associated with FAO [58] (ELS, CE and MEH, unpublished observations). That we could not detect peroxide emission from muscle mitochondria under the same phosphorylating conditions as used here [29] suggests that oxidative inactivation of aconitase or α-KG may not be occurring in the present system. Nevertheless, it is notable that under conditions of high FAO, we observed increased accumulation of malate-citrate and α-KG, a pattern consistent with a saturation or reduced activity of α-KG dehydrogenase activity with increasing FAO. Such a hypothetical mechanism linking FAO and TCA cycle metabolism warrants further examination.

### Methodological considerations

The unique analytical approaches used in conjunction with intact and highly functional mitochondria in the current study have enabled specific analyses of complex mitochondrial processes. An additional advantage of using an enriched mitochondrial preparation instead of whole muscle is that it allows rapid separation of mitochondrial matrix and extra-mitochondrial fractions. It should be noted however that cellular components extrinsic to mitochondria are present in the mitochondrial preparation used here and in numerous published bioenergetic studies. Yet, some extrinsic enzymes and organelles are linked to mitochondria via protein-protein interactions. For example, the endoplasmic reticulum is well known to be functionally and physically linked to mitochondria in liver (*e.g.* [59]) and skeletal muscle [60]. FA elongation occurring in the endoplasmic reticulum may be the source of C>16 FA in the present study. Hexokinase (I and II) is associated with the mitochondrial outer membrane via binding to porin [61]. Binding of hexokinase II to porin on skeletal muscle mitochondria has been reported [62], and we have detected mitochondrial hexokinase activity (ELS, CE and MEH, unpublished). Thus, flux through hexokinase likely accounts for glucose disappearance in the matrix fraction; data from control incubations indicate however that this is unrelated to FA provisioning. Localization of lactic acid dehydrogenase to the inner membrane of skeletal muscle mitochondria has also been reported [63], and would account for lactic acid accumulation in the matrix. It is likely that other enzymes not normally associated with mitochondria will be identified in the future, such as glucose 6-phosphate dehydrogenase [64].

Peroxisomes also participate in β-oxidation, but do not chain-shorten beyond 6 carbons. The acetyl-CoA and medium-chain acyl-CoA species formed by peroxisomal β -oxidation are exported to the cytosol where they are incorporated into biosynthetic pathways or can enter mitochondria and be completely oxidized. Thus it is possible that some of the detected fatty acids were derived from peroxisomes. It is noteworthy, however, that, in the absence of carnitine, O_2_ consumption is not detectable with palmitate concentrations below 200 µM (ELS, CE, MEH, unpublished observations); thus our results are likely to mainly reflect mitochondrial processes. As well, since peroxisomes lack a TCA cycle, the detected TCAi would have strictly originated in mitochondria.

In summary, this ‘proof-of-principle’ mitochondrial metabolomics study highlights that skeletal muscle FAO rate is associated with distinct changes in metabolites within and exported from mitochondria. We also demonstrate that net accumulation and efflux of TCA cycle intermediates is responsive to FAO in skeletal muscle mitochondria. Its extent, and the specific metabolites that are exported, depend on FAO rate. Future studies utilizing targeted metabolomics are required to further characterize and quantify TCA cycle cataplerosis during oxidation of different substrates, at different rates, in skeletal muscle, as well as to better understand anaplerotic mechanisms.

## Supporting Information

Figure S1 14C-Palmitate oxidation rates for the three palmitate conditions tested. Complete (14CO2) and incomplete (14C-ASP) oxidation were determined. Data were re-plotted from Seifert et al., 2008.
(0.13 MB PPT)

Figure S2 Palmitate oxidation rate is associated with unique shifts in TCA cycle intermediates, fatty acids, and amino acids. Presented is a simplified overview of the findings presented in Figures 5–7 and S1, and, for clarity, does not include every pathway and fate of each metabolite. Only the detected TCAi are shown. Low palmitate oxidation rate is associated with the efflux of citrate (panel A). Since matrix levels of citrate do not decrease, anaplerotic replenishment of citrate is occurring. Exogenously provided malate is one likely anaplerotic substrate. Amino acids may be another source. Also effluxed at the low oxidation rate are C12 and C14 fatty acids; these may be derived from β-oxidation reactions, through cleavage of acyl-CoA units by a thioesterase, or may be derived from membrane-bound pools. In contrast (panel B) , elevated palmitate oxidation rate leads to an increase in matrix citrate without export, whereas α-ketoglutarate efflux is prominent. The amino acid pool associated with the matrix fraction diminishes, likely reflecting the anaplerotic replenishment of TCA cycle intermediates. Note that amino acids that are not classically found in the mitochondrial matrix were detected in the matrix fraction; this may reflect pathways that are intimately associated with mitochondria. TCA: tricarboxylic acid; ASP: acid soluble product.
(0.50 MB PPT)

Table S1 Bioenergetic status of murine skeletal muscle mitochondria oxidizing palmitate: Supplementary Table and accompanying Results and Discussion.
(0.05 MB DOC)

Table S2 Complete listing of metabolites detected in each condition according to criteria described in Methods.
(0.16 MB XLS)

Methods S1 Methods for the determination of absolute amounts of metabolites.
(0.03 MB DOC)
