# Supplementary material for: Long-Chain Fatty Acid Combustion Rate Is Associated with Unique Metabolite Profiles in Skeletal Muscle Mitochondria
Source: PLoS One. 2010 Mar 24;5(3):e9834. doi: 10.1371/journal.pone.0009834 (PMC2844415; doi:10.1371/journal.pone.0009834)
Supplement: Figure S1 — 14C-Palmitate oxidation rates for the three palmitate conditions tested. Complete (14CO2) and incomplete (14C-ASP) oxidation were determined. Data were re-plotted from Seifert et al., 2008. (0.13 MB PPT) [file pone.0009834.s001.ppt]

## Slide 1
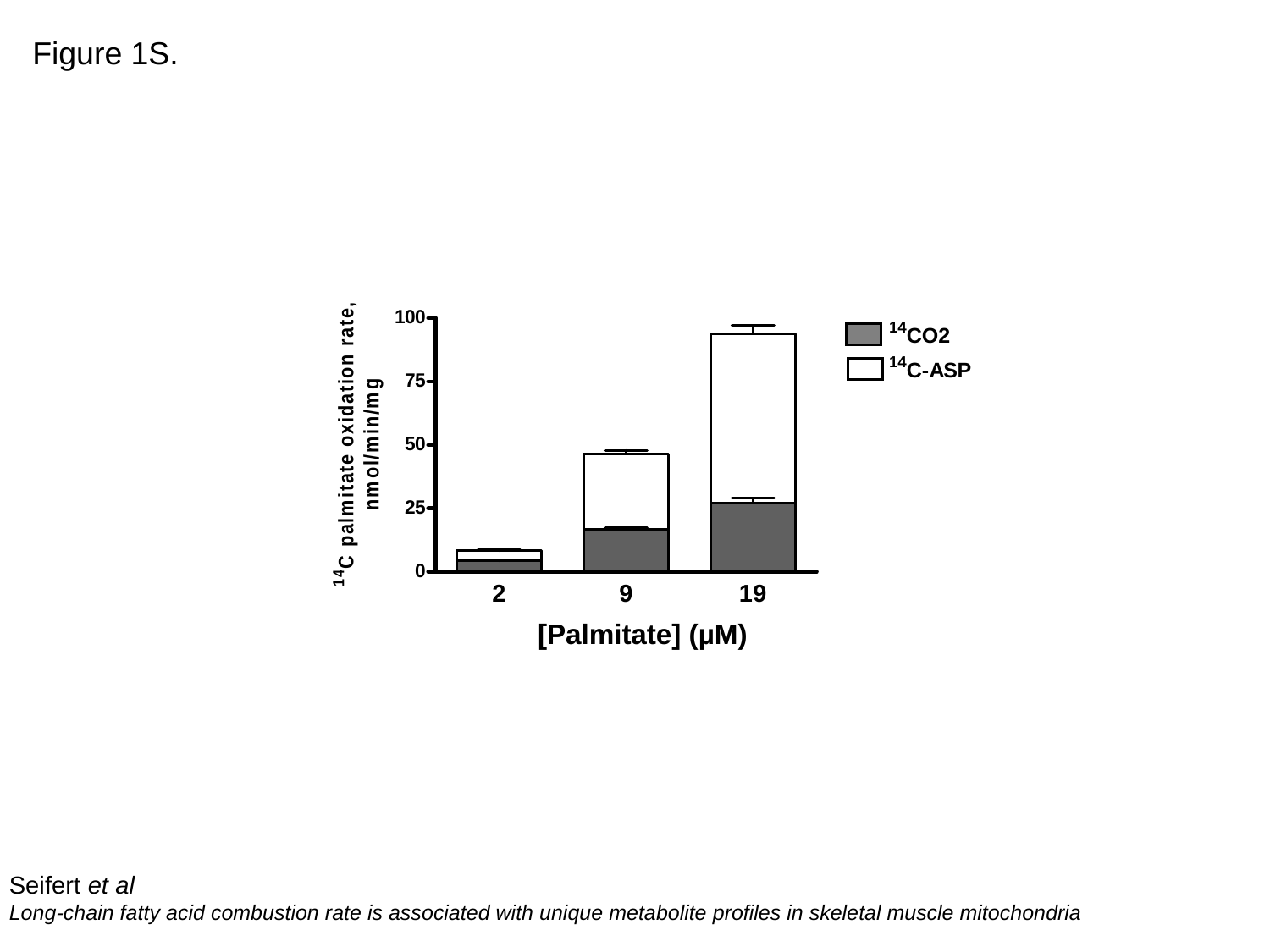

Figure 1S.
[Palmitate] (µM)
Seifert et al
Long-chain fatty acid combustion rate is associated with unique metabolite profiles in skeletal muscle mitochondria
