# Supplementary figures and images for: Long-Chain Fatty Acid Combustion Rate Is Associated with Unique Metabolite Profiles in Skeletal Muscle Mitochondria
Source: PLoS One. 2010 Mar 24;5(3):e9834. doi: 10.1371/journal.pone.0009834 (PMC2844415; doi:10.1371/journal.pone.0009834)

## Slide 1
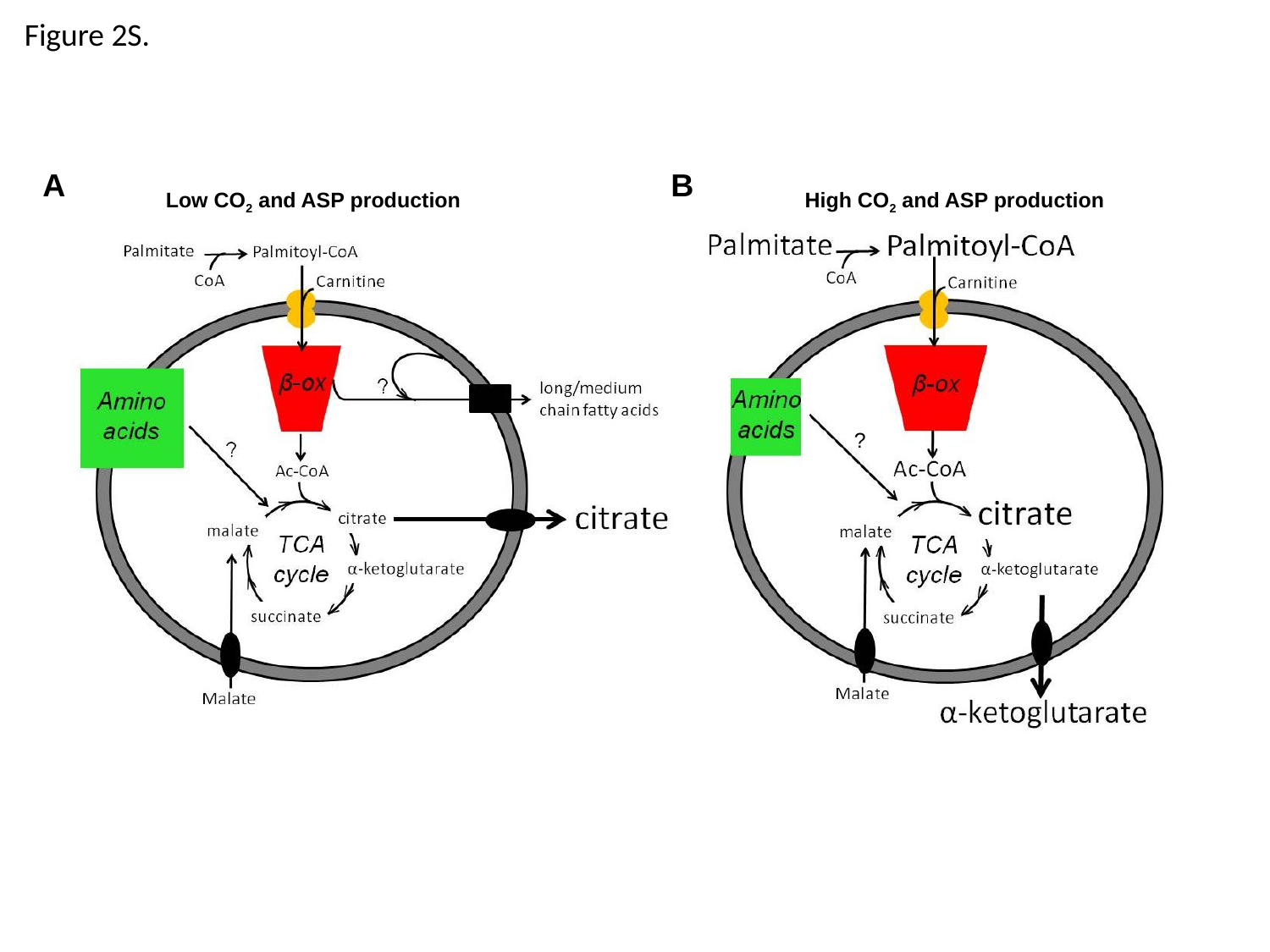

Figure 2S.
A
B
Low CO2 and ASP production
High CO2 and ASP production
?

Supplement: Figure S2 — Palmitate oxidation rate is associated with unique shifts in TCA cycle intermediates, fatty acids, and amino acids. Presented is a simplified overview of the findings presented in Figures 5– 7 and S1, and, for clarity, does not include every pathway and fate of each metabolite. Only the detected TCAi are shown. Low palmitate oxidation rate is associated with the efflux of citrate (panel A). Since matrix levels of citrate do not decrease, anaplerotic replenishment of citrate is occurring. Exogenously provided malate is one likely anaplerotic substrate. Amino acids may be another source. Also effluxed at the low oxidation rate are C12 and C14 fatty acids; these may be derived from β-oxidation reactions, through cleavage of acyl-CoA units by a thioesterase, or may be derived from membrane-bound pools. In contrast (panel B) , elevated palmitate oxidation rate leads to an increase in matrix citrate without export, whereas α-ketoglutarate efflux is prominent. The amino acid pool associated with the matrix fraction diminishes, likely reflecting the anaplerotic replenishment of TCA cycle intermediates. Note that amino acids that are not classically found in the mitochondrial matrix were detected in the matrix fraction; this may reflect pathways that are intimately associated with mitochondria. TCA: tricarboxylic acid; ASP: acid soluble product. (0.50 MB PPT) [file pone.0009834.s002.ppt]
