# Supplementary material for: Long-Chain Fatty Acid Combustion Rate Is Associated with Unique Metabolite Profiles in Skeletal Muscle Mitochondria
Source: PLoS One. 2010 Mar 24;5(3):e9834. doi: 10.1371/journal.pone.0009834 (PMC2844415; doi:10.1371/journal.pone.0009834)
Supplement: Table S1 — Bioenergetic status of murine skeletal muscle mitochondria oxidizing palmitate: Supplementary Table and accompanying Results and Discussion. (0.05 MB DOC) [file pone.0009834.s003.doc]

**Supplementary Table 1S and accompanying Results and Discussion.**

**Table 1S. Bioenergetic state of skeletal muscle mitochondria oxidizing palmitate.**

| **Palmitate oxidation (nmol O/min/mg):** |
| --- |
| **+ ATP + FCCP n** |
| **19 µM Palmitate** 43.5 ± 0.8 55.8 ± 2.1 5 |
| **9 µM** **Palmitate** 30.2 ± 0.7 39.8 ± 1.2 5 |
| **2 µM** **Palmitate** 9.0 ± 0.2 10.3 ± 0.3 5 |
| **Palmitoyl-L-carnitine (PCarn) oxidation (nmol O/min/mg):** |
| **State 3 State 4 RCR n** |
| **19 µM** **PCarn** 87.1 ± 3.1 17.3 ± 0.4 5.1 ± 0.4 3 |
| **9 µM PCarn** 76.2 ± 7.4 17.2 ± 2.4 4.6 ± 1.1 3 |
| **2 µM PCarn** 37.2 ± 2.0 10.3 ± 0.3 3.1 ± 0.5 3 |
| **Pyruvate/malate (P/M; 10 mM/ 5mM) oxidation (nmol O/min/mg):** |
| **State 3 State 4 RCR n** |
| **P/M no free palm** 273.0 ± 0.5 27.8 ± 1.4 9.9 ± 0.3 3 |
| **P/M +19 µM free palm** 261.1 ± 8.0 25.4 ± 1.7 10.3 ± 0.4 3 |

For palmitate oxidation, CoA and ATP were added to activate palmitate activation, and carnitine was added to enable uptake of activated palmitate into mitochondria (see Experimental Procedures). Malate was used to prime the tricarboxylic acid cycle (see text below). ATP hydrolysis to ADP supported phosphorylating oxidation. FCCP: carbonyl cyanide 4-(trifluoromethoxy)phenylhydrazone; State 3: maximal phosphorylating O_2_ consumption; State 4: maximal non-phosphorylating O_2_ consumption; RCR: respiratory control ratio (State 3/State 4); palm: palmitate; n: sample size. Values: mean ± SEM.

**Results and Discussion**

To determine the bioenergetic status of mitochondria oxidizing palmitate, O_2_ consumption was measured using a Clark-type electrode at 37 °C. Incubations were with the same medium and concentration of albumin as for mitochondrial preparations used for metabolomics analyses (see Experimental Procedures). As shown in Table 1S, O_2_ consumption increased with increasing palmitate supply, and, at all [palmitate], exceeded that measured with malate alone (malate + ATP: 5.4 ± 0.6 nmol O/min/mg; used to prime the tricarboxylic acid cycle). Addition of FCCP (carbonyl cyanide 4-(trifluoromethoxy)phenylhydrazone) increased O_2_ consumption by ~25%; thus palmitate was being oxidized at submaximal rates under the incubation conditions used for metabolomics analyses. We next compared these rates to uncoupled oxidation rates. Because oligomycin lowered the rate of ^14^CO_2_ production (not shown), likely due to decreased formation of activated palmitate and thus of oxidizable substrate, we instead evaluated the respiratory control ratio (RCR) of mitochondria oxidizing palmitoyl-L-carnitine as well as the effect of 19 µM palmitate (non-activated) on pyruvate/malate respiration to determine the extent of uncoupling due to free palmitate. We reasoned that the RCR of mitochondria oxidizing palmitoyl-L-carnitine sets an upper limit for the RCR of mitochondria oxidizing palmitate; any uncoupling by free palmitate would lower the RCR. The RCR measured with palmitoyl-L-carnitine was ~ 4. Because addition of 19 µM palmitate had little effect on pyruvate/malate respiration, the RCR of mitochondria oxidizing palmitate is estimated to be ~ 4. In fact, much higher free [palmitate] is required to uncouple skeletal muscle mitochondria as uncoupling could only be detected with [palmitate] > 200 µM [1]. It is also notable that, for a given supply of fatty acid substrate, O_2_ consumption is higher with palmitoyl-L-carnitine than palmitate; this is likely because the concentration of oxidizable substrate is lower with palmitate supply, as we have previously observed [2]. Yet, oxidation rates with palmitate may be closer to rates in intact muscle because much of the control resides at CPT1 which is bypassed when palmitoyl-L-carnitine is supplied as the substrate. Altogether, we conclude that under our incubation conditions, mitochondria oxidize palmitate under phosphorylating conditions, at a rate between state 3 and state 4, but closer to state 3.

References

1. Seifert EL, Estey C, Xuan JY, Harper ME (2009) Electron transport chain dependent and independent mechanisms of mitochondrial H2O2 emission during long-chain fatty acid oxidation. J Biol Chem.

2. Seifert EL, Bezaire V, Estey C, Harper ME (2008) Essential role for uncoupling protein-3 in mitochondrial adaptation to fasting but not in fatty acid oxidation or fatty acid anion export. J Biol Chem 283: 25124-25131.
