# Supplementary material for: Long-Chain Fatty Acid Combustion Rate Is Associated with Unique Metabolite Profiles in Skeletal Muscle Mitochondria
Source: PLoS One. 2010 Mar 24;5(3):e9834. doi: 10.1371/journal.pone.0009834 (PMC2844415; doi:10.1371/journal.pone.0009834)
Supplement: Methods S1 — Methods for the determination of absolute amounts of metabolites. (0.03 MB DOC) [file pone.0009834.s005.doc]

**Supplemental Methods: Quantification of metabolites**

Seven-point calibration curves were generated using identical GC-TOF mass spectrometry conditions as applied for the mitochondria samples (see Experimental Procedures). Pure reference compounds (all purchased from Sigma-Aldrich, St. Louis, MO) were weighed at 10±0.1 mg using a calibrated balance with ±0.01 mg accuracy and dissolved in 10 ml of pure MilliQ water (Millipore, Bellerica, MA) or chloroform/methanol/water (2/5/2) for lipophilic compounds. A geometric dilution series with constant 1:1 ratios between any adjacent dilutions were prepared from these stock solutions. Between each dilution step, tubes were vigorously mixed for 5 s to ensure completely homogenous solution. Appropriate aliquots were taken from each tube into derivatization vials to yield 0.1-25 ug of each of the corresponding reference standards. Solvents were evaporated in a SpeedVac concentrator and derivatized and analyzed by GC-TOF MS as given in the Experimental Procedures. From peak heights using the quantification masses for each compound as defined in the BinBase algorithm protocols, linear equations were calculated starting at peak intensities that had signal/noise levels exceeding 5:1. Parameters of the calibration curves were used for further transformation to molar amounts. Mitochondrial yield from muscle was estimated as mg mitochondrial protein per muscle wet weight obtained from 24 isolations, from mice of a similar age as was used in the present study, performed in our laboratory over the past 2 years; calculated yield was 5.1 ± 0.3 mg mitochondrial protein/muscle wet weight. Average time 0 and delta 20 min peak values for a particular metabolite were subsequently converted from arbitrary units to nmol/mg muscle wet and nmol/mg muscle wet weight/min by using the calibration curve for that metabolite and the estimated mitochondrial yield.
